# Circulating C1q complement/TNF-related protein (CTRP) 1, CTRP9, CTRP12 and CTRP13 concentrations in Type 2 diabetes mellitus: *In vivo* regulation by glucose

**DOI:** 10.1371/journal.pone.0172271

**Published:** 2017-02-16

**Authors:** Bo Bai, Bo Ban, Zunjing Liu, Man Man Zhang, Bee Kang Tan, Jing Chen

**Affiliations:** 1 Neurobiology Institute, Jining Medical University, Jinin, China; 2 Department of Endocrine and Metabolic diseases, Jining Medical College Affiliated Hospital, Jining Medical University, Jining, China; 3 Department of Neurology, China-Japan Friendship Hospital, Beijing, China; 4 Warwick Medical School, University of Warwick, Coventry, United Kingdom; 5 Department of Obstetrics and Gynaecology, Birmingham Heartlands and Solihull Hospitals, Heart of England NHS Foundation Trust, Birmingham, United Kingdom; East Tennessee State University, UNITED STATES

## Abstract

**Objectives:**

The C1q complement/TNF-related protein (CTRP) superfamily, which includes the adipokine adiponectin, has been shown in animal models to have positive metabolic and cardiovascular effects. We sought to investigate circulating CTRP1, CTRP9, CTRP12 and CTRP13 concentrations in persons with type 2 diabetes mellitus (T2DM), with age and BMI matched controls, and to examine the effects of a 2 hour 75g oral glucose tolerance test (OGTT) on serum CTRP1, CTRP9, CTRP12 and CTRP13 levels in persons with T2DM.

**Design:**

Cross-sectional study [newly diagnosed T2DM (n = 124) and control (n = 139) participants]. Serum CTRP1, CTRP9, CTRP12 and CTRP13 were measured by ELISA.

**Results:**

Systolic and diastolic blood pressure, total cholesterol (TCH), Low-density lipoprotein (LDL)-cholesterol, triglycerides, TCH/High-density lipoprotein (HDL) ratio, triglycerides/HDL ratio, glucose, insulin, homeostatic model assessment–insulin resistance (HOMA-IR), C-reactive protein and endothelial lipase were significantly higher, whereas leptin and adiponectin were significantly lower in T2DM participants. Serum CTRP1 were significantly higher and CTRP12 significantly lower in T2DM participants. Age, diastolic blood pressure, glucose and CTRP12 were predictive of serum CTRP1; leptin was predictive of serum CTRP9; glucose and CTRP1 were predictive of serum CTRP12; endothelial lipase was predictive of serum CTRP13. Finally, serum CTRP1 were significantly higher and CTRP12 significantly lower in T2DM participants after a 2 hour 75g OGTT.

**Conclusions:**

Our data supports CTRP1 and CTRP12 as potential novel biomarkers for the prediction and early diagnosis of T2DM. Furthermore, pharmacological agents that target CTRP1 and CTRP12 could represent a new strategy in the treatment of T2DM.

## Introduction

Obesity has been associated with the global pandemic of type 2 diabetes mellitus (T2DM) and cardiovascular diseases (CVDs). Adipose tissue secretes substances called ‘adipokines’ that have been implicated in the pathophysiology of T2DM and CVDs [1–3].

The adipokine adiponectin was the first to be identified in the adipokines of the C1q complement/TNF-related protein (CTRP) superfamily. Adiponectin has anti-diabetic and favorable cardiovascular effects [4]. Raised adiponectin receptors levels have been reported in insulin resistant states [5]. We have reported low circulating concentrations of the adipokine, CTRP3 (also known as cartonectin, cartducin, CORS-26), and its *in vivo* regulation by glucose in humans [6]. We have also reported that metformin significantly increases circulating CTRP3 concentrations in insulin resistant women with PCOS [7]. Other members of the CTRP family have also been identified as adipokines.

CTRP1 is an adipokine predominantly produced by the stromal-vascular cells of adipose tissue, with insulin sensitizing effects [8].

Of all CTRPs identified thus far, CTRP9 has the greatest structural similarity with adiponectin, with its globular C1q domain having the highest degree of amino acid identity with adiponectin [9]. CTRP9 can be secreted as hetero-oligomers with adiponectin [9]. CTRP9 is preferentially produced in adipose tissue, and both adipocytes and stromal-vascular cells produce CTRP9 equally [9]. CTRP9 activates AMPK signaling pathways in cultured myotubes, and adenoviral overexpression of CTRP9 in leptin deficient obese *ob/ob* mice significantly lowers blood glucose levels [9].

CTRP12 (adipolin) is an adipokine [10], with insulin sensitizing and anti-inflammatory effects [10]. In addition, lower circulating and adipose tissue adipolin concentrations were found in rodent models of obesity and diabetes [10]. Like adiponectin, CTRP12 has anti-inflammatory effects i.e. CTRP12 treatment decreases macrophage accumulation and pro-inflammatory gene expression in the adipose tissue of obese mice [10].

CTRP13 is an adipokine, produced mainly by the stromal-vascular cells in adipose tissue and the brain [11]. Also, circulating CTRP13 concentrations are increased in leptin-deficient obese *ob/ob* mice [11]. CTRP13 stimulates glucose transport into cultured adipocytes, hepatocytes and myotubes, and improves fatty acid-induced insulin resistance in cultured hepatocytes by suppressing lipid-induced stress signalling [11].

With the aforementioned in mind, we measured circulating CTRP1, CTRP9, CTRP12 and CTRP13 concentrations in newly diagnosed persons with T2DM as well as age and BMI matched controls. We also examined the effects of a 2 hour 75g oral glucose tolerance test (OGTT) on circulating CTRP1, CTRP9, CTRP12 and CTRP13 concentrations.

## Materials and methods

The study protocol has been described in detail previously [12].

### Ethics

The study was approved by the medical ethics committee of the Affiliated Hospital of Jining Medical University, Shandong, China and written informed consent was obtained from all participants, in accordance with the guidelines in The Declaration of Helsinki 2000.

### Participants

This is a cross-sectional study. One hundred and twenty-four newly diagnosed persons with T2DM (as per the World Health Organization criteria) [13] and one hundred and thirty-eight persons without diabetes participated in this study (Table 1). All study participants had had a random venous plasma glucose concentration, and a value ≥ 11.1 mmol/l designated the participant as having T2DM. Exclusion criteria included a history of congestive heart failure, liver or kidney disease, malignancy, pregnancy and any drugs influencing body weight like corticosteroids or contraceptives. Participants were recruited consecutively (1 January 2013 to 31 December 2014) and were persons attending the Affiliated Hospital of Jining Medical University for screening for diabetes.

**Table 1:** Clinical, hormonal and metabolic features of study subjects; circulating levels of CTRP1, 9, 12 and 13, are derived from overnight fasted patient blood samples.

Variable	T2DM (*n* = 124)	Controls (*n* = 138)	Significance
Sex (women/men)	58/66	64/74	
Age (year)	51.0 (43.0–59.0)	49.0 (41.0–56.0)	NS
BMI (kg/m^2^)	25.8 (22.9–28.4)	25.0 (23.0–27.2)	NS
SBP (mm Hg)	130.0 (120.0–145.0)	121.0 (110.0–128.0)	*P* < 0.01
DBP (mm Hg)	84.5 (75.0–95.0)	75.0 (68.0–83.0)	*P* < 0.01
TCH (mmol/L)	5.1 (4.4–5.8)	4.6 (4.1–5.1)	*P* < 0.01
HDL-cholesterol (mmol/L)	1.3 (1.1–1.6)	1.4 (1.2–1.5)	NS
LDL-cholesterol (mmol/L)	3.0 (2.5–3.5)	2.7 (2.3–3.1)	*P* < 0.01
VLDL-cholesterol (mmol/L)	0.8 (0.5–1.0)	1.0 (0.7–1.0)	NS
Triglycerides (mmol/L)	1.4 (1.0–2.1)	1.0 (0.8–1.3)	*P* < 0.01
TCH/HDL	3.9 (3.3–4.5)	3.4 (3.1–3.8)	*P* < 0.01
TG/HDL	1.2 (0.6–1.7)	0.7 (0.5–1.1)	*P* < 0.01
Glucose (mmol/L)	8.1 (7.1–11.8)	4.9 (4.6–5.3)	*P* < 0.01
Insulin (pmol/L)	63.1 (41.1–94.6)	54.3 (38.3–72.0)	*P* < 0.05
HOMA-IR	3.7 (2.3–5.4)	1.7 (1.2–2.3)	*P* < 0.01
CRP (mg/l)	6.8 (2.4–6.8)	1.8 (0.8–2.5)	*P* < 0.01
Endothelial lipase (ng/ml)	24.7 (18.1–30.6)	18.8 (7.6–32.3)	*P* < 0.01
Leptin (ng/ml)	6.8 (2.4–6.8)	8.0 (3.1–11.4)	*P* < 0.05
Adiponectin (μg/ml)	11.9 (6.7–16.5)	15.8 (11.1–25.6)	*P* < 0.01
CTRP1 (ng/ml)	543.3 (430.8–708.8)	308.7 (255.2–354.9)	*P* < 0.01
CTRP9 (ng/ml)	149.0 (108.2–194.0)	135.4 (111.4–154.0)	NS
CTRP12 (pg/ml)	446.8 (387.7–506.4)	808.5 (753.8–892.4)	*P* < 0.01
CTRP13 (ng/ml)	201.1 (149.3–240.0)	198.0 (160.5–223.3)	NS

Data are median (interquartile range). Group comparison by Mann-Whitney *U* test. BMI = body mass index; SBP = systolic blood pressure; DBP = diastolic blood pressure; TCH = total cholesterol; HDL = High-density lipoprotein; LDL = Low-density lipoprotein; VLDL = Very low-density lipoprotein; TG = triglycerides; HOMA-IR = homeostatic model assessment–insulin resistance; HbA_1C_ = glycated haemoglobin; CRP = C-reactive protein; CTRP = C1q complement/TNF-related protein; NS = not significant.

After the initial random blood glucose test, all study participants were requested to fast overnight, fasting blood samples were collected and immediately centrifuged in all 262 study participants. A 2 hour 75g OGTT was also performed in participants with T2DM. Serum was immediately aliquoted on ice and stored at -80°C. All participants underwent anthropometric measurements. Blood pressure was measured in a sitting position within a quiet and calm environment after a rest of at least 5 minutes. The average of three measurements was obtained. Blood samples and data collection were performed by B.Bai, B.Ban and M.M.Z.

### Biochemical and hormonal analysis

Assays for glucose, HbA1c (only in T2DM participants), total cholesterol (TCH), high-density lipoprotein (HDL)-cholesterol, low-density lipoprotein (LDL)-cholesterol, very low-density lipoprotein (VLDL)-cholesterol and triglycerides (TG) [Hitachi 7600 biochemical automatic analyser] as well as for insulin (Roche electrochemiluminescence analyser) were performed. The estimate of insulin resistance by homeostatic model assessment–insulin resistance (HOMA-IR) score was calculated as Io (μIU/mL) x Go (mmol/L)/22.5, where Io is the fasting insulin (μIU/mL) and Go (mmol/L) is the fasting glucose, as described by Matthews et al. [14] The TCH/HDL and TG/HDL ratios were calculated as indices of ischaemic heart disease mortality and morbidity [15,16]. C-reactive protein (CRP) concentrations in sera were measured using a commercially available ELISA kit (Aviscera, Santa Clara, USA; Catalog number: SK00080-01) according to manufacturer’s protocol, with an intra-assay coefficient of variation of less than 6%. Endothelial lipase concentrations in sera were measured using a commercially available ELISA kit (Cloud-Clone Corp, Houston, USA; Catalog number: SEA469Hu) according to manufacturer’s protocol, with an intra-assay coefficient of variation of less than 10%. Leptin concentrations in sera were measured using a commercially available ELISA kit (Aviscera, Santa Clara, USA; Catalog number: SK00050-02) according to manufacturer’s protocol, with an intra-assay coefficient of variation of less than 8%. Total adiponectin concentrations in sera were measured using a commercially available ELISA kit (Biovision, Milpitas, USA; Catalog number: K4901-100) according to manufacturer’s protocol, with an intra-assay coefficient of variation of less than 8%. CTRP1 concentrations in sera were measured using a commercially available ELISA kit (BioVendor, Inc., Czech Republic; Catalog number: RD191153100R) according to manufacturer’s protocol, with an intra-assay coefficient of variation of 2.7%. CTRP9 concentrations in sera were measured using a commercially available ELISA kit (USCN Life Science, Inc., Wuhan, China; Catalog number: SER877Hu) according to manufacturer’s protocol, with an intra-assay coefficient of variation of less than 10%. CTRP12 concentrations in sera were measured using a commercially available ELISA kit (Aviscera, Santa Clara, USA; Catalog number: SK00392-06) according to manufacturer’s protocol, with an intra-assay coefficient of variation of less than 8%. CTRP13 concentrations in sera were measured using a commercially available ELISA kit (Aviscera, Santa Clara, USA; Catalog number: SK00333-06) according to manufacturer’s protocol, with an intra-assay coefficient of variation of less than 6%.

### Statistical analysis

There were no missing data. Non-parametric statistical analyses were employed. Data were analysed by Mann-Whitney *U* test and Wilcoxon matched pairs test. Data are medians (interquartile range). Spearman Rank correlation was used for calculation of associations between variables. Subsequently, if individual bivariate correlations achieved statistical significance, variables were entered into a linear regression model and multiple regression analysis was performed. All statistical analyses were performed using SPSS version 24.0 (SPSS, Inc.). P < 0.05 was considered significant.

## Results

Table 1 shows the anthropometric, biochemical and hormonal parameters in all participants. Systolic Blood Pressure (SBP), Diastolic Blood Pressure (DBP), TCH, LDL-cholesterol, TG, TCH/HDL ratio, TG/HDL ratio, glucose, insulin, HOMA-IR, CRP and endothelial lipase were significantly higher, whereas leptin and adiponectin were significantly lower in T2DM participants; 22 out of the 138 controls and 54 out of the 124 T2DM study participants had hypertension i.e. either SBP ≥ 140mmHg and/or DBP ≥ 90mmHg. All of these 76 study participants were newly diagnosed hypertensives (diagnosed at the time of the study) and thus none of them were on any anti-hypertensive medications. Furthermore, T2DM study participants had median HbA1c: 66.1mmol/mol (8.2%); interquartile range: 53.0mmol/mol (7.0%) to 85.8mmol/mol (10.0%).

Serum CTRP1 concentrations were significantly higher in participants with T2DM compared to controls [543.3 (430.8–708.8) vs. 308.7 (255.2–354.9)ng/ml; P < 0.01: Table 1]. Serum CTRP12 concentrations were significantly lower in participants with T2DM compared to controls [446.8 (387.7–506.4) vs. 808.5 (753.8–892.4)pg/ml; P < 0.05: Table 1]. Serum CTRP9 and serum CTRP13 concentrations were not significantly different (Table 1).

Furthermore, in all participants (*n* = 262), women had higher serum leptin [7.2 (4.4–12.7) vs. 4.6 (1.5–7.6)n/ml; P < 0.01] and serum adiponectin [16.5 (11.1–25.6) vs. 12.2 (8.3–16.5)μg/ml; P < 0.01] concentrations compared to men; however, there were no significant difference with respect to serum CTRP1 [364.3 (278.9–543.3) vs. 386.3 (302.7–550.8)ng/ml; P > 0.05], serum CTRP9 [134.5 (108.4–169.5) vs. 138.0 (112.8–180.5)ng/ml; P > 0.05], serum CTRP12 [726.4 (428.7–852.0) vs. 558.8 (412.9–816.9)pg/ml; P > 0.05] and serum CTRP13 [198.0 (135.3–233.3) vs. 198.0 (168.0–220.5)ng/ml; P > 0.05] concentrations.

### Effects of a 2 hour 75g OGTT in T2DM participants on serum CTRP1, CTRP9, CTRP12 and CTRP13 concentrations

Glucose and insulin concentrations were significantly higher in T2DM participants after a 2 hour 75g OGTT (Table 2).

**Table 2:** Effects of a 2 hour 75g OGTT on serum glucose, insulin, CTRP1, CTRP9, CTRP12 and CTRP13 concentrations in T2DM subjects (n = 124).

	0 hours	2 hours	Significance
Glucose (mmol/L)	8.1 (7.1–11.8)	14.9 (12.1–19.2)	*P* < 0.01
Insulin (pmol/L)	63.1 (41.1–94.6)	231.7 (172.3–360.5)	*P* < 0.01
CTRP1 (ng/ml)	543.3 (430.8–708.8)	689.8 (538.5–847.5)	*P* < 0.01
CTRP9 (ng/ml)	149.0 (108.2–194.0)	150.5 (64.9–235.5)	NS
CTRP12 (pg/ml)	446.8 (387.7–506.4)	282.1 (256.5–308.2)	*P* < 0.01
CTRP13 (ng/ml)	201.1 (149.3–240.0)	205.3 (136.5–261.3)	NS

Data are median (interquartile range). Group comparison by Wilcoxon matched pairs test.

On the other hand, serum CTRP1 concentrations were significantly higher, and serum CTRP12 concentrations were significantly lower in T2DM participants after a 2 hour 75g OGTT (Table 2). Serum CTRP9 and serum CTRP13 concentrations were not significantly different (Table 2).

### Correlation of CTRP1, CTRP9, CTRP12 and CTRP13 with covariates

In all participants (*n* = 262), Spearman Rank analysis showed that serum CTRP1 levels were significantly correlated with age, SBP, DBP, TCH, LDL-cholesterol, TG, TCH/HDL ratio, TG/HDL ratio, glucose, insulin, HOMA-IR, CRP, leptin, adiponectin and CTRP12 (Table 2). When subjected to multiple regression analysis, age (*β* = 0.118; *P =* 0.043), DBP (*β* = 0.188; *P =* 0.021), glucose (*β* = 0.456; *P* < 0.010) and CTRP12 (*β* = -0.153; *P* < 0.010) were predictive of serum CTRP1 levels (Table 3).

**Table 3:** Linear regression analysis of variables associated with CTRP1 (*n* = 262).

	Simple		Multiple	
Variable	*r*	*P*	*β*	*P*
Age (year)	0.140	**0.024**	0.118	**0.043**
BMI (kg/m^2^)	0.021	0.731		
SBP (mm Hg)	0.271	**< 0.010**	-0.061	0.454
DBP (mm Hg)	0.311	**< 0.010**	0.188	**0.021**
TCH (mmol/L)	0.173	**< 0.010**	0.006	0.972
HDL-cholesterol (mmol/L)	-0.075	0.227		
LDL-cholesterol (mmol/L)	0.129	**0.037**	-0.104	0.192
VLDL-cholesterol (mmol/L)	0.012	0.841		
Triglycerides (mmol/L)	0.287	**< 0.010**	0.440	0.294
TCH/HDL	0.223	**< 0.010**	0.119	0.333
TG/HDL	0.244	**< 0.010**	-0.317	0.416
Glucose (mmol/L)	0.596	**< 0.010**	0.456	**< 0.010**
Insulin (pmol/L)	0.125	**0.044**	0.376	0.082
HOMA-IR	0.411	**< 0.010**	-0.414	0.080
CRP (mg/l)	0.382	**< 0.010**	0.077	0.186
Endothelial lipase (ng/ml)	0.092	0.138		
Leptin (ng/ml)	-0.148	**0.017**	-0.094	0.086
Adiponectin (μg/ml)	-0.230	**< 0.010**	-0.024	0.664
CTRP9 (ng/ml)	0.084	0.176		
CTRP12 (pg/ml)	-0.383	**< 0.010**	-0.153	**< 0.010**
CTRP13 (ng/ml)	0.026	0.673		

Spearman Rank correlation was used for calculation of associations between variables. If individual bivariate correlations achieved statistical significance, multiple regression analysis with CTRP1 was performed to test the joint effect of these parameters on CTRP1. BMI = body mass index; SBP = systolic blood pressure; DBP = diastolic blood pressure; TCH = total cholesterol; HDL = High-density lipoprotein; LDL = Low-density lipoprotein; VLDL = Very low-density lipoprotein; TG = triglycerides; HOMA-IR = homeostatic model assessment–insulin resistance; CRP = C-reactive protein; CTRP = C1q complement/TNF-related protein; NS = not significant.

In all participants (*n* = 262), Spearman Rank analysis showed that serum CTRP9 concentrations were significantly correlated with SBP, CRP and leptin (Table 4). When subjected to multiple regression analysis, leptin (*β* = 0.181; *P* < 0.010) was predictive of serum CTRP9 concentrations (Table 4).

**Table 4:** Linear regression analysis of variables associated with CTRP9 (*n* = 262).

	Simple		Multiple	
Variable	*r*	*P*	*β*	*P*
Age (year)	0.007	0.913		
BMI (kg/m^2^)	0.054	0.386		
SBP (mm Hg)	0.122	**0.049**	0.065	0.315
DBP (mm Hg)	0.072	0.247		
TCH (mmol/L)	-0.070	0.259		
HDL-cholesterol (mmol/L)	-0.039	0.525		
LDL-cholesterol (mmol/L)	-0.026	0.679		
VLDL-cholesterol (mmol/L)	-0.011	0.865		
Triglycerides (mmol/L)	0.093	0.133		
TCH/HDL	0.013	0.828		
TG/HDL	0.082	0.187		
Glucose (mmol/L)	0.050	0.420		
Insulin (pmol/L)	0.045	0.471		
HOMA-IR	0.092	0.139		
CRP (mg/l)	0.125	**0.044**	0.073	0.254
Endothelial lipase (ng/ml)	0.076	0.220		
Leptin (ng/ml)	0.129	**0.038**	0.181	**< 0.010**
Adiponectin (μg/ml)	0.004	0.943		
CTRP1 (ng/ml)	0.084	0.176		
CTRP12 (pg/ml)	-0.049	0.427		
CTRP13 (ng/ml)	0.049	0.425		

Spearman Rank correlation was used for calculation of associations between variables. If individual bivariate correlations achieved statistical significance, multiple regression analysis with CTRP9 was performed to test the joint effect of these parameters on CTRP9. BMI = body mass index; SBP = systolic blood pressure; DBP = diastolic blood pressure; TCH = total cholesterol; HDL = High-density lipoprotein; LDL = Low-density lipoprotein; VLDL = Very low-density lipoprotein; TG = triglycerides; HOMA-IR = homeostatic model assessment–insulin resistance; CRP = C-reactive protein; CTRP = C1q complement/TNF-related protein; NS = not significant.

In all participants (*n* = 262), Spearman Rank analysis showed that serum CTRP12 levels were significantly correlated with TG, TG/HDL ratio, glucose, HOMA-IR, CRP, adiponectin and CTRP1 (Table 5). When subjected to multiple regression analysis, glucose (*β* = -0.253; *P* < 0.010) and CTRP1 (*β* = -0.155; *P =* 0.023) were predictive of serum CTRP12 levels (Table 5).

**Table 5:** Linear regression analysis of variables associated with CTRP12 (*n* = 262).

	Simple		Multiple	
Variable	*r*	*P*	*β*	*P*
Age (year)	0.084	0.173		
BMI (kg/m^2^)	0.001	0.985		
SBP (mm Hg)	-0.057	0.357		
DBP (mm Hg)	-0.118	0.056		
TCH (mmol/L)	-0.060	0.333		
HDL-cholesterol (mmol/L)	0.025	0.693		
LDL-cholesterol (mmol/L)	-0.045	0.465		
VLDL-cholesterol (mmol/L)	-0.064	0.299		
Triglycerides (mmol/L)	-0.168	**< 0.010**	0.001	0.995
TCH/HDL	-0.078	0.207		
TG/HDL	-0.134	**0.030**	0.078	0.730
Glucose (mmol/L)	-0.489	**< 0.010**	-0.253	**< 0.010**
Insulin (pmol/L)	-0.108	0.008		
HOMA-IR	-0.333	**< 0.010**	-0.092	0.157
CRP (mg/l)	-0.211	**< 0.010**	-0.072	0.231
Endothelial lipase (ng/ml)	-0.049	0.431		
Leptin (ng/ml)	0.078	0.209		
Adiponectin (μg/ml)	0.212	**< 0.010**	-0.033	0.585
CTRP1 (ng/ml)	-0.383	**< 0.010**	-0.155	**0.023**
CTRP9 (pg/ml)	-0.049	0.427		
CTRP13 (ng/ml)	0.049	0.425		

Spearman Rank correlation was used for calculation of associations between variables. If individual bivariate correlations achieved statistical significance, multiple regression analysis with CTRP12 was performed to test the joint effect of these parameters on CTRP12. BMI = body mass index; SBP = systolic blood pressure; DBP = diastolic blood pressure; TCH = total cholesterol; HDL = High-density lipoprotein; LDL = Low-density lipoprotein; VLDL = Very low-density lipoprotein; TG = triglycerides; HOMA-IR = homeostatic model assessment–insulin resistance; CRP = C-reactive protein; CTRP = C1q complement/TNF-related protein; NS = not significant.

In all participants (*n* = 262), Spearman Rank analysis showed that serum CTRP13 concentrations were significantly correlated with BMI, TG, TG/HDL ratio and endothelial lipase (Table 6). When subjected to multiple regression analysis, endothelial lipase (*β* = 0.171; *P* < 0.010) was predictive of serum CTRP13 concentrations (Table 6).

**Table 6:** Linear regression analysis of variables associated with CTRP13 (*n* = 262).

	Simple		Multiple	
Variable	*r*	*P*	*β*	*P*
Age (year)	0.074	0.231		
BMI (kg/m^2^)	0.124	**0.046**	0.068	0.267
SBP (mm Hg)	0.025	0.685		
DBP (mm Hg)	0.049	0.427		
TCH (mmol/L)	-0.012	0.849		
HDL-cholesterol (mmol/L)	-0.070	0.260		
LDL-cholesterol (mmol/L)	-0.018	0.766		
VLDL-cholesterol (mmol/L)	-0.041	0.511		
Triglycerides (mmol/L)	0.126	**0.041**	-0.222	0.356
TCH/HDL	0.086	0.163		
TG/HDL	0.155	**0.012**	0.351	0.144
Glucose (mmol/L)	0.033	0.595		
Insulin (pmol/L)	0.021	0.735		
HOMA-IR	0.042	0.495		
CRP (mg/l)	0.092	0.136		
Endothelial lipase (ng/ml)	0.181	**< 0.010**	0.171	**< 0.010**
Leptin (ng/ml)	-0.036	0.565		
Adiponectin (μg/ml)	0.056	0.366		
CTRP1 (ng/ml)	0.026	0.673		
CTRP9 (pg/ml)	0.049	0.425		
CTRP12 (ng/ml)	0.093	0.132		

Spearman Rank correlation was used for calculation of associations between variables. If individual bivariate correlations achieved statistical significance, multiple regression analysis with CTRP13 was performed to test the joint effect of these parameters on CTRP13. BMI = body mass index; SBP = systolic blood pressure; DBP = diastolic blood pressure; TCH = total cholesterol; HDL = High-density lipoprotein; LDL = Low-density lipoprotein; VLDL = Very low-density lipoprotein; TG = triglycerides; HOMA-IR = homeostatic model assessment–insulin resistance; CRP = C-reactive protein; CTRP = C1q complement/TNF-related protein; NS = not significant.

## Discussion

This is the first study to report circulating CTRP1, CTRP9, CTRP12 and CTRP13 levels, simultaneously, in newly diagnosed persons with T2DM (*n* = 124) as well as age and BMI matched controls (*n* = 138), a total of 262 participants. Clinical studies have demonstrated that circulating CTRP1 levels are significantly positively associated with fasting blood glucose and HOMA-IR [18–20]. We found that serum CTRP1 levels were significantly higher in participants with T2DM and significantly associated with age, TCH, glucose, HOMA-IR and adiponectin, consistent with the current literature [17–19]. Of relevance, Rodriguez et al., using a CTRP1 knockout mice model, found that loss of CTRP1 resulted in perturbations of glucose and lipid metabolism [20]. In addition, we report novel significant associations of serum CTRP1 with SBP, DBP, TG, TCH/HDL ratio, TG/HDL ratio, insulin, CRP, leptin and CTRP12. Once again, in keeping with existing research reports [17–19], we show that age and glucose were predictive of serum CTRP1 concentrations; furthermore, we report for the first time that DBP and CTRP12 were predictive of serum CTRP1 levels; the case can be vice-versa as CTRP1 has been shown to stimulate aldosterone production and could lead to hypertension [21]. This increase of circulating CTRP1 concentrations may be a compensatory response to insulin resistance akin to that of leptin resistance; the increase could also reflect a CTRP1 resistant state. However, there may be other yet undetermined factors that may account for the significantly higher circulating CTRP1 levels found in T2DM participants.

In mice, circulating levels of CTRP9 are low in obese mice (fed with a high-fat diet); overexpression of CTRP9 produced lean mice that were resistant to weight gain [22]. Furthermore, CTRP9 knockout mice exhibited an obese phenotype when fed with standard laboratory chow [23]. In humans, conflicting results have been reported in relation to circulating CTRP9 concentrations with metabolic dysfunction; Hwang *et al*. observed lower serum CTRP9 levels in older persons with metabolically unhealthy profiles and lower serum total adiponectin levels compared to persons with higher CTRP9 levels [24] whereas Wolf *et al*. found that serum CTRP9 concentrations were higher in obese compared to lean [25] persons and serum CTRP9 levels decreased following weight loss surgery [25]. Furthermore, Jung *et al*. reported a significant positive association with HOMA-IR and brachial ankle pulse wave velocity (baPWV) but no association with serum adiponectin levels in persons with T2DM [26]. We found that serum CTRP9 concentrations in T2DM participants were lower but this was not statistically significant. However, we did find that serum CTRP9 levels were significantly correlated with SBP, CRP and leptin, and that leptin was predictive of serum CTRP9 levels.

Circulating and adipose tissue CTRP12 levels have been shown to be lower in insulin resistant women with PCOS, and that metformin treatment leads to higher circulating and adipose tissue CTRP12 levels [27,28]. We present novel data of lower CTRP12 concentrations in newly diagnosed persons with T2DM compared to age and BMI matched controls. Moreover, serum CTRP12 levels were significantly correlated with TG, TG/HDL ratio, glucose, HOMA-IR, CRP, adiponectin and CTRP1, of these, glucose and CTRP1 were found to be predictive of serum CTRP12 levels. Therefore, low CTRP12 concentrations reflect insulin resistance. Of clinical pharmacological relevance, Wei et al. showed that increasing CTRP12 circulating levels in both obese and diabetic mice enhanced insulin sensitivity and lowered blood glucose concentrations [29].

We did not find a significant difference in serum CTRP13 concentrations between newly diagnosed persons with T2DM and age and BMI matched controls. Nevertheless, we found that serum CTRP13 concentrations were significantly correlated with BMI, TG, TG/HDL ratio and endothelial lipase and that endothelial lipase was predictive of serum CTRP13 levels; however, whether CTRP13 regulates the expression of endothelial lipase or vice-versa remain to be clarified.

Furthermore, we found that serum adiponectin concentrations were significantly lower in T2DM compared to control participants, in agreement with the current research literature [30]. In addition, we had observed significantly lower leptin levels in our newly diagnosed T2DM participants [median HbA1c: 66.1mmol/mol (8.2%); interquartile range: 53.0mmol/mol (7.0%) to 85.8mmol/mol (10.0%)]. This is consistent with previous studies reporting lower leptin levels in poorly controlled persons with T2DM [31].

We also show novel data that serum CTRP1 concentrations were significantly higher, and serum CTRP12 concentrations were significantly lower in T2DM participants after a 2 hour 75g OGTT, however, there were no significant differences in serum CTRP9 and serum CTRP13 concentrations. Both glucose and insulin levels were significantly higher in T2DM participants after the 2 hour 75g OGTT. Thus, glucose and/or insulin could account for our observations as they have been shown to regulate adipokines [32,33]. Given our findings that glucose was strongly predictive for serum CTRP1 (*β* = 0.456; *P* < 0.010) and serum CTRP12 (*β* = -0.253; *P* < 0.010) but not insulin, we postulate that alterations in glucose metabolism would mainly account for our findings.

A novel data is the inverse relationship between serum CTRP1 with serum CTRP12 levels, and importantly in multivariate analyses, serum CTRP12 was found to be predictive of serum CTRP1 levels and *vice versa*. As mentioned in the introduction section, CTRP1 is predominantly produced by the stromal-vascular cells whereas CTRP12 by adipocytes in adipose tissue. Could CTRP1 in stromal-vascular cells regulate the production of CTRP12 in adipocytes? On the same token, leptin was noted to predict serum CTRP9 concentrations, suggesting that leptin may regulate the production of CTRP9, plausibly in adipose tissue. Additionally, endothelial lipase was observed to be determinative of serum CTRP13 levels, which is the first evidence in humans to suggest a connection between CTRP13 and vascular disease; endothelial lipase have been shown to play an important role in the development of atherosclerosis [34]. Future studies should address these questions, which at present, is beyond the remit of this study.

Considering the limitations of our study, although the diets of all study participants were perceived to be similar as they were all from the local catchment area of the Affiliated Hospital of Jining Medical University, this was not formally assessed and could be a confounding factor in our findings. In addition, our study only involved a Chinese population.

In conclusion, CTRP1 and CTRP12 could serve to predict as well as to diagnose T2DM. Moreover, drugs that target CTRP1 and CTRP12 could benefit patients with T2DM.
